# Bioethics in Childbirth Care: Protocol for a Scoping Review

**DOI:** 10.2196/29921

**Published:** 2021-07-26

**Authors:** Graziani Izidoro Ferreira, Kevin Haley Barbosa, Andre Di Carlo Duarte, Cesar De Oliveira, Dirce Guilhem

**Affiliations:** 1 University of Brasilia Brasilia Brazil; 2 University Centre of Brasília Brasilia Brazil; 3 UNIEURO University Centre Brasilia Brazil; 4 University College London London United Kingdom

**Keywords:** childbirth care, bioethics, scoping review, childbirth, parturition, labor

## Abstract

**Background:**

Ensuring women’s rights during childbirth care based on humanized and bioethical principles results in better quality of care and patient safety and provides positive childbirth experiences.

**Objective:**

We aim to explore the available evidence on the application of bioethical principles in the general context of childbirth care.

**Methods:**

Our scoping review will follow the Joanna Briggs Institute Reviewer’s Manual. Published and unpublished bibliographic materials will be considered based on the following inclusion criteria: reports of the application of bioethical principles (concept) in assistance to the predelivery, childbirth, and postpartum periods (population) in the hospital context (context). We will search for relevant studies in PubMed and the Virtual Health Library, including MEDLINE, LILACS, BDENF, SCiELO, Web of Science, and Google Scholar. Two reviewers will perform the screening of titles and abstracts, read the full texts, and extract data from the selected articles. The data will then be organized and expressed into categories based on their content.

**Results:**

The analyzed data will be presented through flowcharts, tables, and descriptive narratives. A paper summarizing the findings from this review will be published in a peer-reviewed journal. In addition, a synthesis of the key findings will be disseminated to health services linked to university hospitals in Brazil. They will also be shared with the academic community and policy makers involved in the Childbirth Assistance Network, which will potentially adopt our recommendations in their decision-making process regarding childbirth care practice in Brazil.

**Conclusions:**

The findings from this review will inform, through the translation of knowledge, childbirth support groups, feminist movements, movements in favor of humanization of childbirth, and other childbirth support networks in the country.

**Trial Registration:**

Open Science Framework; https://osf.io/kczyr/

## Introduction

### Background

In 2014, the World Health Organization (WHO) released a statement on the abuse and disrespect that many women suffer during childbirth globally. Professionals in the field and researchers have frequently demonstrated patterns of mistreatment and disrespect for women during childbirth. These patterns, conceptualized as obstetric violence in many countries [1], have varied from psychological and physical aggression such as verbal abuse to neglect and abandonment, humiliation and punishment, and coercive and forced care.

The WHO’s declaration shows the real dimension of these ill-treatments, that is, the violation of human rights. Health and human rights in maternal law should always reflect the bioethical issues that involve childbirth assistance, especially in doctor-patient conflicts and the abusive use of technologies that can make the risk greater than the benefit [1].

The high morbidity and mortality rates made the search for respectful and better-quality care necessary [2]. Thus, the goals of a sustainable development aimed at reducing these rates have been sought since the implementation of the millennium goals in 2003 [3]. The increasing changes toward the promotion and protection of maternal rights in the clinical and care scenarios should be accompanied by reflections on bioethics and human rights in childbirth [4].

### Research Question and Objective

In this context, we used the acronym PCC (population, concept, and context), defined below, to elaborate the research question:

- P (population): women in labor, delivery, and the postpartum period
- C (concept): bioethics and health care
- C (context): childbirth care in the hospital context

Thus, the following question was raised: “Do women in labor, delivery, and the postpartum period have their rights ensured, through health care based on bioethics during care?”

Based on this research question, the main objective of this review is to map the available evidence on the application of bioethical principles in the general context of childbirth care.

Many studies have addressed bioethical issues during childbirth care. However, after conducting a search with the descriptors “bioethics,” “ethics,” “birth,” and “childbirth” in the Joanna Briggs Institute Evidence-Based Practice Database, Cochrane Library, and PubMed, no systematic reviews, overviews, or scoping overviews were found in this context. Only one systematic review was found in the Web of Science database that addresses the effectiveness of respectful care during childbirth and birth services, but it does not address bioethical issues.

## Methods

### Study Design

To achieve the proposed objective in accordance with the research question, we decided to use the scoping review methodology, which is a type of systematic review, mapping concepts and findings related to the topic of interest available in the main data sources by using the knowledge synthesis approach [5]. The scoping review uses all types of evidence at various levels.

### Protocol

This scoping review will be conducted according to the guidelines and methodological framework published by the Joanna Briggs Institute for scoping reviews. The expected development time and duration of the study is 6 months.

Prior to the elaboration of this protocol, a search of review articles already published on the topic to be studied was carried out, as described below.

### Search Strategy

The search strategy (Table 1) covered the following aspects:

- Type of studies: systematic review, overview, and scoping review
- Descriptors: bioethics and midwifery; bioethics and (delivery or “natural childbirth” or labor or obstetric or parturition or “perinatal care”); “human rights” and childbirth
- Filters: year of publication (last 10 years), type of study

**Table 1:** Review articles identified in electronic databases.

Search strategy	Joanna Briggs Institute Evidence-Based Practice Database	PubMed	Cochrane	Web of Science	Health Systems Evidence	Google Scholar	Findings	Excluded	Met the inclusion criteria
Bioethics and childbirth	1	5	0	12	0	200 early^a^	218	218	0
“Human rights” and childbirth	3	20	1	89	2	200 early	315	315	0
Bioethics and (delivery or “natural childbirth” or labor or obstetric or parturition or “perinatal care”)	6	382	11	169	0	8	576	576	0
Bioethics and midwifery	3	39	0	7	0	200 early	249	249	0

^a^For searches performed on Google Scholar, we analyzed the first 200 articles due to the large number of random findings.

### Inclusion Criteria

#### Participants

Studies that included participants who are women; are of any age; and received labor, delivery, and postpartum care in any hospital, maternity, or delivery house will be included in the review. Studies that included participants who received care during home birth will be excluded.

#### Concept

The central concept of the included studies is that they should address bioethical issues. They should include details related to bioethical principles; feminist-inspired bioethics; principled theory; or any bioethical or human, maternal, or women's rights foundation.

#### Context

The context to be observed should be assistance during labor, delivery, and postpartum, regardless of the country, state, city, or region. The abuses and mistreatment of women during childbirth care as a global issue is important, as well as different perspectives from different locations need to be considered. Only hospital birth care services are of interest in terms of the context of the research question in this review.

#### Study Types

The source of information will remain open to allow the inclusion of any type of study. All studies will undergo a methodological quality assessment, using the Joanna Briggs Institute Critical Appraisal Tools, with the scores described in tables next to the description of the selected articles.

#### Period

Studies published in the last 10 years, developed in any year and duration, will be eligible for the review.

#### Language

Full articles published in English, Portuguese, and Spanish will be considered for inclusion.

## Results

### Information Sources and Search Strategy

#### First Stage

An initial search was performed in two electronic databases, MEDLINE (PubMed) and Virtual Health Library. After this initial search, words contained in the title, summary or abstract, and keywords of the articles found were analyzed. The descriptors found will be compared to the descriptors registered in the descriptor bases: Medical Subject Headings (MeSH) for searches in PubMed and DeCS (Descriptores en Ciencias de la Salud [Health Sciences Descriptors]) for searches to be carried out in the Virtual Health Library (Table 2).

**Table 2:** Descriptors selected for search strategy.

DeCS^a^				MeSH^b^
Portuguese	English	Spanish		
Bioética	Bioethics	Bioética	Bioethics	
Ética Médica	Ethics	Ética Médica	Ethics	
Direitos Humanos	Human Rights	Derechos Humanos	Human rights	
Autonomia	Autonomy	Autonomía Personal	Autonomy	
Beneficência	Beneficence	Beneficencia	Beneficence	
Não maleficência	Non-maleficence	No maleficencia	Non-maleficence	
Justiça	Justice	Justicia	Justice	
Eficiência	Efficiency	Eficiencia	Efficiency	
Proporcionalidade	Proportionality	Proporcionalidad	Proportionality	
Obstetrícia	Obstetric	Obstetricia	Obstetric	
Parto	Childbirth	Parto	Childbirth; parturition; labour; labor	
Parto Normal	Natural childbirth	Parto Normal	Delivery; perinatal care; natural childbirth	
Assistência ao Parto	Midwifery	Partería	Midwifery	

^a^DeCS: Descriptores en Ciencias de la Salud (Health Sciences Descriptors).

^b^MeSH: Medical Subject Headings.

#### Second Stage

A second search using all identified keywords and descriptors will be carried out in all databases: PubMed, Virtual Health Library, SCiELO, and Web of Science, following the search strategy described in Table 3.

**Table 3:** Search strategy across different databases.

Database	Search strategy
PubMed	("bioethics"[MeSH Terms] OR "bioethics"[All Fields]) AND (("delivery, obstetric"[MeSH Terms] OR ("delivery"[All Fields] AND "obstetric"[All Fields]) OR "obstetric delivery"[All Fields] OR "delivery"[All Fields]) OR "natural childbirth"[All Fields] OR ("labour"[All Fields] OR "work"[MeSH Terms] OR "work"[All Fields] OR "labor"[All Fields] OR "labor, obstetric"[MeSH Terms] OR ("labor"[All Fields] AND "obstetric"[All Fields]) OR "obstetric labor"[All Fields]) OR obstetric[All Fields] OR ("parturition"[MeSH Terms] OR "parturition"[All Fields] OR "delivery, obstetric"[MeSH Terms] OR ("delivery"[All Fields] AND "obstetric"[All Fields]) OR "obstetric delivery"[All Fields]) OR "perinatal care"[All Fields]) ([Midwifery OR Natural Childbirth OR Childbirth OR Obstetric) AND Autonomy[All Fields]) (Midwifery OR Natural Childbirth OR Childbirth OR Obstetric) AND Beneficence (Midwifery OR Natural Childbirth OR Childbirth OR Obstetric) AND Non-maleficence (Midwifery OR Natural Childbirth OR Childbirth OR Obstetric) AND Justice (Midwifery OR Natural Childbirth OR Childbirth OR Obstetric) AND Efficiency (Midwifery OR Natural Childbirth OR Childbirth OR Obstetric) AND Proportionality
Web of Science	[bioethics and (delivery or “natural childbirth” or labor or obstetric or parturition or “perinatal care”)] or ["human rights" and childbirth] (Midwifery OR Natural Childbirth OR Childbirth OR Obstetric) AND Beneficence (Midwifery OR Natural Childbirth OR Childbirth OR Obstetric) AND Non-maleficence (Midwifery OR Natural Childbirth OR Childbirth OR Obstetric) AND Justice (Midwifery OR Natural Childbirth OR Childbirth OR Obstetric) AND Efficiency (Midwifery OR Natural Childbirth OR Childbirth OR Obstetric) AND Proportionality
SCiELO	bioethics and (delivery or “natural childbirth” or labor or obstetric or parturition or “perinatal care”) "human rights" and childbirth (Midwifery OR Natural Childbirth OR Childbirth OR Obstetric) AND Beneficence (Midwifery OR Natural Childbirth OR Childbirth OR Obstetric) AND Non-maleficence (Midwifery OR Natural Childbirth OR Childbirth OR Obstetric) AND Justice (Midwifery OR Natural Childbirth OR Childbirth OR Obstetric) AND Efficiency (Midwifery OR Natural Childbirth OR Childbirth OR Obstetric) AND Proportionality
BVS^a^	bioética and ("assistência ao parto" or nascimento or parto) "direitos humanos" and ("assistência ao parto" or nascimento or parto) autonomia and ("assistência ao parto" or nascimento or parto) (Midwifery OR Natural Childbirth OR Childbirth OR Obstetric) AND Beneficence (Midwifery OR Natural Childbirth OR Childbirth OR Obstetric) AND Non-maleficence (Midwifery OR Natural Childbirth OR Childbirth OR Obstetric) AND Justice (Midwifery OR Natural Childbirth OR Childbirth OR Obstetric) AND Efficiency (Midwifery OR Natural Childbirth OR Childbirth OR Obstetric) AND Proportionality
Google Acadêmico (Google Scholar)	bioética and "assistência ao parto" bioética and parto direitos humanos and "assistência ao parto" (Midwifery OR Natural Childbirth OR Childbirth OR Obstetric) AND Beneficence (Midwifery OR Natural Childbirth OR Childbirth OR Obstetric) AND Non-maleficence (Midwifery OR Natural Childbirth OR Childbirth OR Obstetric) AND Justice (Midwifery OR Natural Childbirth OR Childbirth OR Obstetric) AND Efficiency (Midwifery OR Natural Childbirth OR Childbirth OR Obstetric) AND Proportionality

^a^BVS: Biblioteca Virtual en Salud (Virtual Health Library).

#### Third Stage

A third search will be carried out in the grey literature through Google Scholar. Thus, the search will be complete in five different databases. If necessary, the list of references used in all articles selected from the full text and included in the review will be consolidated. We will contact authors of primary studies or reviews to obtain more information about published studies, if relevant. All these steps and care will be undertaken to maximize the scope of the search and the scope of the important studies to be considered in this review. Thus, articles published in any language will be considered eligible if they meet the remaining eligibility criteria.

### Selection of Studies

The following steps will be undertaken to select published articles based on prespecified inclusion criteria: (1) screening by year of publication, (2) sorting by title, (3) screening by summary or abstract, and (4) full-text screening.

The selection process of the studies to be included will be carried out by two independent researchers. Doubts or divergences will be resolved through consensus, or a third researcher will be called for a final decision regarding the inclusion or exclusion of a study.

### Data Extraction

The important information that will be mapped is as follows: article title, year of publication, study objective, population or sample, study design, main conclusions, and key findings related to the scoping review’s research question.

Records that identify each study will be preserved, should further checks be required. In the course of data extraction, it may become important to add unanticipated information that will be additional if useful to help answer the review question. Thus, the data table will be continuously updated by the research team. The data to be extracted will be tested by one or two team members to make sure that all relevant results are extracted.

### Summary of Results

The results of this scoping review will be presented in a diagram or table as well as in a descriptive format. Analyses of the general data of the included studies will also be presented as graphs or tables, indicating the distribution of the studies by year or period of publication, countries of origin, and research methods. A narrative summary will accompany the results displayed in graphs or tables, describing how the results of each included study are related to the objective and question of this review. Research gaps found and possible limitations of this review will also be highlighted.

## Discussion

### Implications

The main aim of this review is to explore the available evidence on the application of bioethical principles in the general context of childbirth care. We believe that having a clearer picture of how women in labor, delivery, and the postpartum period have their rights ensured through health care based on bioethics, as well as investigating what further actions have been suggested, is critical to addressing the problem of ensuring these women’s rights.

The conclusions from this scoping review will inform decision-makers, health services, and health professionals on bioethics in childbirth care. The findings will also be shared with the entire academic community and public policy makers involved with the Childbirth Assistance Network in Brazil. This, in turn, may result in the implementation of decision-making regarding a safe and humanized childbirth care practice, based on ethical and bioethical principles and ensuring the rights of women.

### Dissemination

The summary of the results will be disseminated through the publication of data in a free-of-charge format and through the translation of knowledge to women, childbirth support groups, feminist movements, movements in favor of the humanization of childbirth, and birth and support networks. We will use social networks and electronic correspondence, in addition to presentations at congresses and scientific events and publication in peer-reviewed scientific journals.

### Conclusions

The strengths of this study relate to a very comprehensive bibliographic search of various databases, electronic sources for difficult-to-locate and unpublished studies (or the grey literature), and publications in any language following Joanna Briggs Institute's methodologically rigorous manual. We will also use different strategies to disseminate our results widely. As a potential limitation, we could cite the time needed to evaluate a large number of articles related to assistance and bioethics.

## Abbreviations

DeCS: Descriptores en Ciencias de la Salud [Health Sciences Descriptors]
MESH: Medical Subjects Headings
WHO: World Health Organization
